# Whole-Brain Dynamics Disruptions in the Progression of Alzheimer’s Disease: Understanding the Influence of Amyloid-Beta and Tau

**DOI:** 10.1101/2024.03.29.587333

**Published:** 2024-03-31

**Authors:** Gustavo Patow, Anira Escrichs, Petra Ritter, Gustavo Deco, for the Alzheimer’s Disease Neuroimaging Initiative

**Affiliations:** 1 ViRVIG, Universitat de Girona, Girona, Catalonia, Spain; 2 Computational Neuroscience Group, Center for Brain and Cognition, Department of Information and Communication Technologies, Universitat Pompeu Fabra, Barcelona, Catalonia, Spain; 3 Berlin Institute of Health at Charité, Universitätsmedizin Berlin, Berlin, Germany; 4 Institució Catalana de la Recerca i Estudis Avançats (ICREA), Barcelona, Catalonia, Spain

**Keywords:** Alzheimer’s Disease, whole-brain dynamics, Amyloid beta, tau, resting-state fMRI, metastability, integration, dynamical complexity

## Abstract

**INTRODUCTION:**

Alzheimer’s disease (AD) affects brain structure and function along its evolution, but brain network dynamic changes remain largely unknown.

**METHODS:**

To understand how AD shapes brain activity, we investigated the spatiotemporal dynamics and resting state functional networks using the intrinsic ignition framework, which characterizes how an area transmits neuronal activity to others, resulting in different degrees of integration. Healthy participants, MCI, and AD patients were scanned using resting state fMRI. Mixed effects models were used to assess the impact of ABeta and tau, at the regional and whole-brain levels.

**RESULTS:**

Dynamic complexity is progressively reduced, with Healthy participants showing higher metastability (i.e., a more complex dynamical regime over time) than observed in the other stages, while AD subjects showed the lowest.

**DISCUSSION:**

Our study provides further insight into how AD modulates brain network dynamics along its evolution, progressively disrupting the whole-brain and resting state network dynamics.

## 1 Background

Neurodegeneration is the progressive loss of structure or function of neurons, which may ultimately involve cell death [26]. Alzheimer’s Disease (AD) is a neurodegenerative disease that affects cortical and subcortical areas of the brain, affecting first the medial temporal lobe and the limbic system, and most areas of the neocortex at later stages [5, 27, 46]. The disease can remain asymptomatic for years but ultimately leads to progressive impairment of memory and other cognitive domains, neuropsychiatric symptoms, and, ultimately, severe impairment in all body functions. This results in a huge loss of quality of life for affected people and caregivers and high societal costs.

Minor cognitive deficits with little influence on daily living are defined as mild cognitive impairment (MCI). In the typical disease course, the deficits extend later to other cognitive domains, such as speech and spatial orientation. When the cognitive impairment is severe enough to affect daily living, the disease is usually defined as dementia (due to AD) [29]. Neurodegeneration can be found in the brain at many different neuronal circuitry levels, from molecular to systemic. These diseases are considered incurable because there is no known way to reverse the progressive degeneration. Diagnoses are subpar, with a 20% misdiagnosis ratio, and better methods are needed for various clinical diagnoses [2]. Some studies [31] suggest that patients with Mild Cognitive Impairment (MCI) to a lesser degree, and dementia (AD), show less variation in neuronal connectivity during resting state, supporting the notion that dynamic functional connectivity (FC) and related measures have the potential of being used as biomarkers of AD.

Resting-state fMRI enables exploring the brain’s intrinsic organization of large-scale distributed networks. Recent neuroimaging studies on Alzheimer’s disease have revealed stage-dependent brain activity fluctuations in several resting-state networks, including the default mode network (DMN), salience network (SN), dorsal attention (DAN), and limbic networks (LN) [3, 6, 18, 22, 25, 33, 35, 38, 41]. To date, only a few fMRI studies have used a whole-brain dynamic approach and showed the impact of Alzheimer’s disease on brain dynamics and information processing across large-scale brain networks [30, 39, 48]. This could provide a deeper understanding of how disease progression alters the underlying brain dynamics, which is critical for providing a more reliable understanding of how AD-related changes impact cognition, emotion, and behavior and for developing targeted interventions for AD-related disorders [17, 45].

The intrinsic ignition framework [10] presents a view of the brain as a complex system characterized by dynamical properties essential for efficient information processing. This framework highlights the significance of intrinsic ignition, which denotes the capacity of brain regions to initiate and sustain neural activity that can propagate throughout the whole brain network, as assessed through the measurement of metastability [11]. Metastability, in this context, refers to the local degree of functional variability of each brain area over time. Notably, this framework has demonstrated remarkable robustness in capturing differences in whole-brain dynamics across different brain states, encompassing both health and disease, such as deep sleep, meditation, aging, depression, and abnormal development [12, 14, 15, 28, 32]. Understanding the underlying whole-brain dynamics is crucial for examining the effects of factors that impact brain function, such as neurodegeneration.

In this study, we aimed to investigate the dynamical complexity of Alzheimer’s disease by examining resting state activity at different levels of analysis, including global, network, and local brain activity patterns in a sample of 36 subjects scanned using fMRI. Specifically, we computed the intrinsic ignition framework [10] across the whole-brain network and within eight well-known resting-state networks (control, DMN, dorsolateral attention, limbic, motor, salience, subcortical, and visual) along three disease phases (healthy controls, MCI, and AD). Furthermore, we employed multilevel modeling to examine the effects of protein burdens, Amyloid-Beta (ABeta), and tau, on brain network dynamics. We hypothesize that misfolded proteins can substantially influence the metastability of whole-brain and resting-state network dynamics.

## 2 Methods

### 2.1 Participants

We used the ADNI database to gather 17 healthy controls (HC), 9 mild cognitive impairment (MCI) patients, and 10 patients with Alzheimer’s Disease (AD) from ADNI, which are mostly the same participants as those used by Stefanovski et al. [42] and Triebkorn et al. [44]. See Table 1.

Given these groups, we used the G Power [16] software to conduct statistical power calculations based on a two-group Wilcoxon-Mann-Whitney test, with significance level *α* = 0.05 and power 1 – *β* = 0.8. Assuming a standard deviation *σ* = 0.05 (a reasonable assumption given our results below), we obtained that the minimum effect size we could discern in this setting would be *d* = 1.1.

### 2.2 Data Acquisition and Processing

As previously mentioned, the images used in this study were downloaded from ADNI-3, which used data from Siemens scan. As this data is the same as previously reported [42], we will limit ourselves to an abridged processing description. The following imaging modalities were included:

- T1 MPRAGE. TE = 2.95– 2.98ms, TR = 2.3 s, matrix and voxel size differ slightly.
- FLAIR. TE differs slightly, TR= 4.8 s, matrix size = 160 × 256 × 256, and voxel size differs slightly.
- DWI (only for 15 HC participants to create an average healthy SC). TE = 56–71ms, TR = 3.4–7.2 s, matrix size = 116 · 116 · 80, voxel size = 2 · 2 · 2, bvals = [0, 1000] or [0, 500, 1000, 2000], bvecs = 49 or 115.
- Siemens Fieldmaps and PET Data (AV-45 for ABeta). The preprocessing of imaging data can be subdivided into structural images, DWI, and PET.

### 2.3 Structural MRI

For each included participant, we created a brain parcellation for our structural data using FLAIR, following the minimal preprocessing pipeline [20] of the Human Connectome Project (HCP) using Freesurfer^1^ [36], FSL [24, 40, 47] and connectome workbench^2^. Therefore, we used T1 MPRAGE, FLAIR, and fieldmaps for the anatomical parcellation. This consists of a Prefreesurfer, Freesurfer, and Postfreesurfer part. Also, the MNI templates were used at 1mm resolution instead of 0.7mm. In the Freesurfer pipeline, all intermediate steps were performed with the original image resolution. We then registered the subject cortical surfaces to the parcellation of Glasser et al. [19] using the multimodal surface matching (MSM) tool [37]. For the registration, we used cortical thickness, myelin maps, cortical curvature, and sulci from the subject and template surface. We mapped the parcellation on the surface back into the gray matter volume with connectome workbench. This volume parcellation surfed as the mask for the connectome and PET intensity extraction. There were 379 regions in this parcellation: 180 left and 180 right cortical regions, 9 left and 9 right subcortical regions, and 1 brainstem region.

### 2.4 fMRI Pre-Processing

The pre-processing of resting-state fMRI was computed using FSL FEAT and independent component analysis–based denoising (FSLFIX) following a standard pipeline [42], which included removal of the first four volumes, rigid-body head motion correction, 3-mm spatial smoothing to improve signal-to-noise ratio, and a high-pass temporal filter of 75 s to remove slow drifts. The data were then denoised using FSLFIX, an independent component analysis–based denoising method that uses an automated classifier to identify noise-related components for removal from the data. The algorithm was trained on a manually labeled held out set of 25 individuals scanned with identical imaging parameters. Using ordinary least squares regression, time courses for noise-labeled components and 24 head motion parameters were removed from the voxel-wise fMRI time series.

The resulting denoised functional data were spatially normalized to the International Consortium for Brain Mapping 152 template in Montreal Neurological Institute (MNI) space using Advanced Normalization Tools (version 2.2.0), via a three-step method: (I) registration of the mean realigned functional scan to the skull-stripped high-resolution anatomical scan via rigid-body registration; (II) spatial normalization of the anatomical scan to the MNI template via a nonlinear registration; and (iii) normalization of the functional scan to the MNI template using a single transformation matrix that concatenates the transforms generated in steps I and II. Mean time series for each parcellated region were then extracted and filtered in the range 0.01 to 0.09 Hz.

### 2.5 Amyloid Beta and tau

In Alzheimer’s disease, one of the hallmarks of the evolution is the accumulation of the peptide amyloid-*β* (ABeta), and the protein tau is a gradual process that involves the accumulation, modification, and assembly of monomeric forms into larger misfolded structures that eventually form fibrillar inclusions. This process is thought to both drive and initiate AD. In Figure±5, we can see that their levels, as measured by PET, systematically increase along the evolution of the disease, often being used as biomarkers for the level of disease progression. In our case, for ABeta, we used the version of AV-45 PET already preprocessed by ADNI, using a standard image with a resolution of 1.5mm cubic voxels and matrix size of 160 × 160 × 96, normalized so that the average voxel intensity was 1 and smoothed out using a scanner-specific filter function and later averaged for the Glasser parcellation. For tau, we also used ADNI’s preprocessed version of AV-1451 (Flortaucipir) following the same acquisition and processing, resulting in a single relative tau value for each voxel, which was also later averaged for the corresponding parcellation values.

### 2.6 Intrinsic Ignition Framework

**Figure 1:**
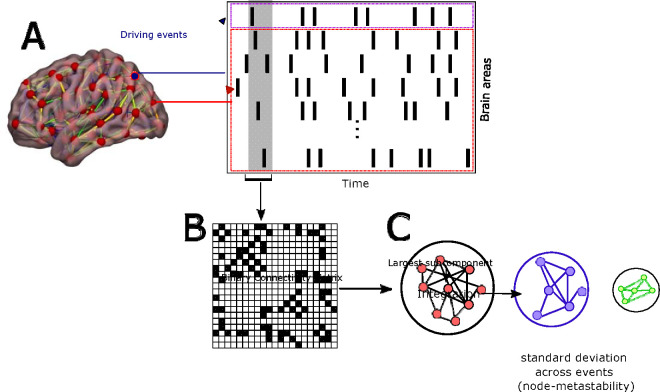
Intrinsic ignition Framework. **(A)** Events were captured applying a threshold method [43] (see purple area). For each event elicited (a gray area), the activity in the rest of the network was measured in the time window of 4TR (see red area). **(B)** A binarized matrix was obtained, representing the connectivity between brain areas where activity was simultaneous. **(C)** Applying the global integration measure [13], we obtained the largest subcomponent. Repeating the process for each driving event, we calculated the node-metastability computed as the standard deviation of the integration of each brain area over time. Figure adapted from [9, 14, 15].

We used the intrinsic-ignition framework [9, 10] to examine the dynamical complexity across three groups (HC, MCI, and AD). The framework assesses the degree of whole-brain integration based on spontaneously occurring events over time. The methodology for calculating intrinsic integration values across brain areas is illustrated in Figure 1. The algorithm involves identifying driving events for each brain area, which are converted into a binary signal using a threshold [43]. To represent events as a binary signal, the time series are transformed into z-scores, denoted as z_i_(t), and a threshold value, θ, is applied. Specifically, an event is marked as 1 in the binary sequence *σ*(t) if *z*_*i*_(t) surpasses the threshold from below and marked as 0 otherwise. Upon triggering an event, the neural activity is measured in all brain areas within a time window of 4TR. Then, a binary matrix is constructed to depict the connectivity between brain areas exhibiting simultaneous activity. The measure of global integration [13] is then computed to assess the broadness of communication across the network for each driving event (i.e., the largest subcomponent). This process is iterated for each spontaneous neural event to obtain the node-metastability, quantified as the standard deviation of the integration for each brain area in the brain network. We computed the framework across the whole-brain network and within eight large-scale networks, i.e., control, DMN, dorsolateral attention, limbic, motor, salience, subcortical, and visual networks.

**Figure 2:**
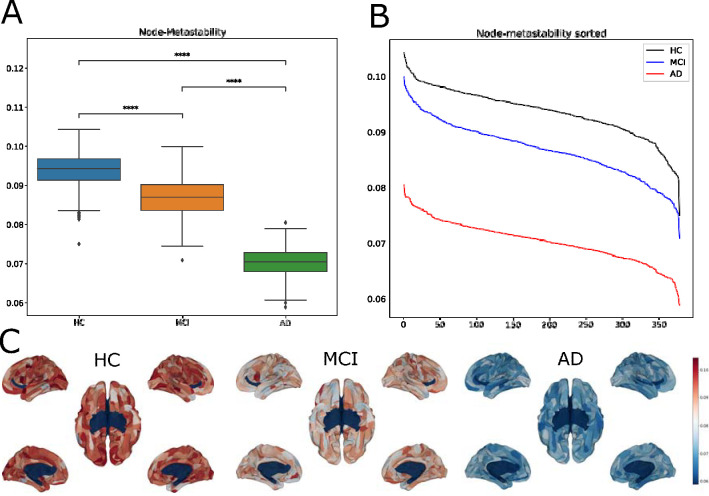
Dynamical complexity of Alzheimer’s Disease stages. **(A)** Node-metastability. Healthy controls showed higher node-metastability values across the whole-brain network than the MCI and AD stages. P-values are based on a two-sample t-test, where **** represents *p <*= 1.00*−e* 04. **(B)** Hierarchy. The red area marks the ten regions showing the highest metastability values in each stage. For the Healthy controls, brain areas showing the highest values were primarily located in the visual, somatomotor, and dorsal attention networks. For the MCI stage, the brain areas belonged to the same networks, except that, besides the visual network, with a lower metastability. Finally, during the AD stage, they were located again in the visual, somatomotor, and dorsal attention networks, although with a severe decrease in metastability. **(C)** Brain renders represent the metastability values of the 379 areas for each disease stage. The dynamical complexity of the healthy controls across the whole-brain networks is more complex than the dynamical complexity of the other two stages. (p-value annotation legend: ns: *p <*= 1.00*e* + 00, *: 1.00*e −* 02 *< p <*= 5.00*e −* 02, **: 1.00*e −* 03 *< p <*= 1.00*e −* 02, ***: 1.00*e −* 04 *< p <*= 1.00*e −* 03, ****: *p <*= 1.00*e −* 04).

We have also defined the Hierarchy Disruption Factor (HDF), which measures the $l^{2}$ norm between two hierarchies, as:

$$HDF(a,b)=\surd \{\sum |a-b{|}^{\wedge }2\}$$

where a and b are two cohort-based (i.e., HC, MCI, or AD) hierarchies. A hierarchy for a cohort is defined by computing, for each node in the parcellation, the averaged metastability over all subjects in that cohort, and then sorted from largest to smallest values [9]. See Figure 2B.

### 2.7 Statistical Analyses

For the metastability analysis, we implemented a Linear Mixed Effects model (LME) using the *lmer* function in R Statistical Software (v4.3.2) [34]) with the lme4 package [4]. We first used the ABeta and tau SUVR values for each region, the patient ID, and the MMSE group for each patient (i.e., HC, MCI, or AD) as explanatory variables. Later, we extended these models to include the effects of ABeta and tau levels on node-metastability within resting state networks, both to refine our analysis and to mirror the studies performed in the rest of the paper.

As mentioned, we first assessed whether the outcome variable (i.e., each node’s metastability) shows a significant change by specifying the node ABeta and tau levels, as well as their interaction, as fixed effects, and the participant ID (subject) as random effect. The interaction between cohort (i.e., disease progression) and amyloid and tau status was added as a fixed effect to the above models to assess whether ABeta and tau accumulation differentially affect change over time. Observe that we only have 3 levels for the cohort, thus it is not recommended to pose it as a random effect [21]. We defined the syntax for the models as *Meta ABeta Tau Cohort* + (1*|ID*).

Then, in the second stage, we repeated the previous analysis considering the RSN for each node as a grouping variable, both as a random and as a fixed effect, to assess the effect of the proteins over each RSN. It is worth mentioning that, in this second stage, the addition of the cohort as a fixed (or even as a random) effect did not provide significant differences, and thus it was not included in the final assessment of the model. The final syntax for this model is *Meta ABeta Tau RSN* + (1 + *RSN|ID/RSN* ).

## 3 Results

### 3.1 Node-metastability Across the Whole-brain Network

We computed the node-metastability measure to study the dynamical complexity underlying the whole-brain functional network in the three groups, i.e., HC (avg=0.094, stdev=0.0044), MCI (avg=0.087, stdev=0.0049) and AD (avg=0.070, stdev=0.0037). We found that the node-metastability significantly decreased in the AD group compared to the MCI (FDR-corrected, *p* < 0.001, effect size *d* = 3.80) and HC (FDR-corrected, *p* < 0.001, effect size *d* = 5.79). Furthermore, we found that the node-metastability was higher in HC than in MCI (FDR-corrected, *p* < 0.001), effect size *d* = 1.45). Observe that, in all the cases, the difference between the average values is larger than the calculated minimum effect size we established before, of *d* = 1.1, given our sample size.

Figure 2A shows the results of this analysis, where we can see a clear, statistically significant difference between the three groups. As we can see, the overall dynamical complexity systematically decreases as the disease progresses, which is to be expected as the different regions have their dynamics altered.

In Figure 2B, we can see the hierarchy for each disease stage across the whole-brain functional network (i.e., the sorted brain areas from highest to lowest node-metastability). The red shadow area represents the 10% brain areas showing the highest node-metastability values for each group. These regions correspond mainly to the visual network, closely followed by regions in the somatomotor and dorsal networks. However, they show a systematic decrease as the disease progresses along its known stages. Our HDF measure showed a clear difference when comparing the cohort-averaged values: *HDF* (*HC, MCI*) = 0.13 and *HDF* (*HC, AD*) = 0.46. However, the measures had too much variance at the individual level to get statistically significant results.

In Figure 2C, we show the rendered brains representing the node-metastability for each group across the whole-brain functional network. Healthy controls show the highest metastability values compared to MCI and AD stages (FDR-corrected, *p* < 0.001).

### 3.2 Node-metastability Across Resting State Networks

**Figure 3:**
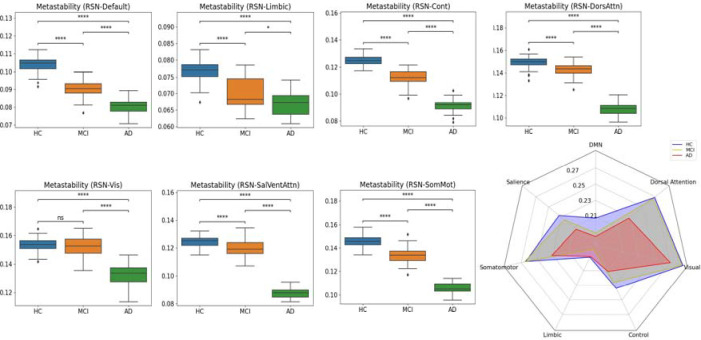
Node-metastability within resting state networks. **(A)** Compared to healthy control subjects, node-metastability was preserved for the visual network between HC and MCI, significantly decreasing in the MCI and AD stages for all the other networks. In general, dorsal attention and somatomotor, although strongly affected, were the ones that more or less preserved their significance with respect to their metastability. P-values are based on a two-sample t-test, where * denotes 1.00*−e* 02 *< p <*= 5.00*e −* 02, and **** denotes *p <*= 1.00*−e* 04. **(B)** The radar plot represents the average metastability values per resting state network for each stage. (p-value annotation legend: ns: *p <*= 1.00*e*+00, *: 1.00*e−*02 *< p <*= 5.00*e−*02, **: 1.00*e−*03 *< p <*= 1.00*e−*02, ***: 1.00*e−*04 *< p <*= 1.00*e−*03, ****: *p <*= 1.00*e −* 04).

We assessed the node-metastability within each network to test group differences across resting-state networks, i.e., control, limbic, somatomotor, salience, DMN, and dorsal. Differences between groups for each network are depicted in Figure 3A. Compared to healthy controls, the MCI group presented a significant decrease in node-metastability across all resting state networks (FDR-corrected, *p* < 0.001) except in the visual network, which remained almost unchanged (FDR-corrected, *p >* 0.05). Furthermore, the AD group showed a significant decrease in node-metastability across all resting-state networks compared to healthy controls (FDR-corrected, *p* < 0.001). Similarly, when compared to MCI, the AD group displayed a node-metastability decline in all networks, i.e., the control, somatomotor, salience, DMN, and dorsal (FDR-corrected, *p* < 0.001) and limbic (FDR-corrected, *p* < 0.05). Figure 3B shows a radar plot illustrating each group’s average metastability values for each resting-state network.

**Figure 4:**
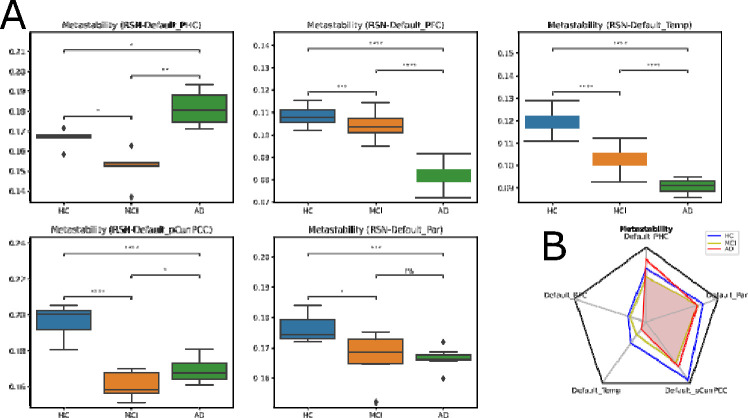
Node-metastability within the default mode network, for each stage of the disease. **(A)** Compared to healthy control subjects, node-metastability was preserved for the PFC sub-network between HC and MCI, and somewhat for the Par subnetwork, which also continued mildly affected in AD. However, Node-metastability significantly decreased in the MCI and AD stages for all the other networks, while showing an increase in AD for PHC, probably due to compensatory mechanisms. P-values are based on a two-sample t-test, where * denotes 1.00*e −* 02 *< p <*= 5.00*e−* 02, and **** denotes *p <*= 1.00*−e* 04. **(B)** The radar plot represents the average metastability values for each stage, per each subregion of the default RSN. (p-value annotation legend: ns: *p <*= 1.00*e*+00, *: 1.00*e−*02 *< p <*= 5.00*e−*02, **: 1.00*e−*03 *< p <*= 1.00*e−*02, ***: 1.00*e−*04 *< p <*= 1.00*e−*03, ****: *p <*= 1.00*e −* 04).

Although these are interesting results in themselves, the literature usually focuses on the behavior of the DMN. To shed further light on this aspect, we repeated the analysis above focused on the DMN subregions: parahippocampal cortex (PHC), prefrontal cortex (PFC), temporal (Temp), precuneus posterior cingulate cortex (pCunPCC), and parietal (Par). The results are presented in Figure 4. As we can see, the disease progression affects the metastability in most subregions in the DMN. In contrast, others (i.e., PHC) show uneven behavior, and some (i.e., pCunPCC) even show an important rise in their metastability values, probably due to some compensatory mechanism.

### 3.3 Multilevel Modeling of AD on Resting State Networks

As mentioned, we used mixed effects models for the whole brain to study the effect of both burdens, ABeta and tau, on the observed metastability. In this study, the metastability for each node was defined as the outcome variable, while, as explanatory variables, we used the ABeta and tau SUVR values for each region, and the patient ID as a random effect. As a parallel of the research done in this paper, we studied two different models, the first one analyzing the influence of both burdens on global metastability, to understand the effect of both misfolded proteins on the whole brain; and the second one including the RSN of each node, to understand their impact on the different networks. In these analyses, we did not consider the disease MMSE stage, as adding it resulted in a more complex model without any significant improvement in its prediction power, as measured with pairwise ANOVA tests.

#### Whole brain:

**Figure 5:**
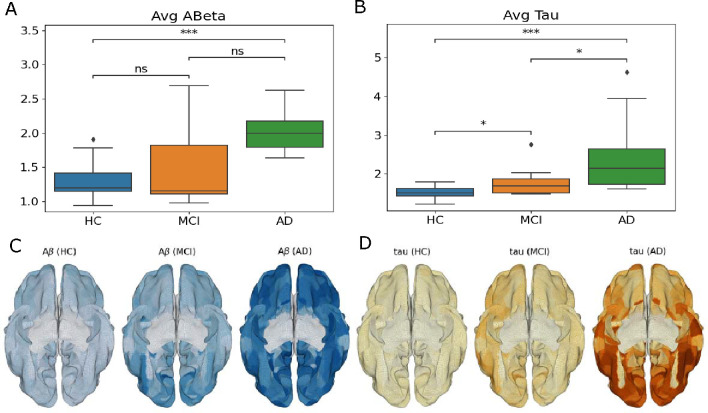
Average per-subject levels of ABeta (A) and Tau (B) for the different AD groups. As we can see, there is a clear increase of both burdens along the disease evolution, which will certainly affect the analyzed metastability values. The lower row illustrates the average values for each cohort group (C, D). (p-value annotation legend: ns: *p <*= 1.00*e* + 00, *: 1.00*e −* 02 *< p <*= 5.00*e −* 02, **: 1.00*e −* 03 *< p <*= 1.00*e −* 02, ***: 1.00*e −* 04 *< p <*= 1.00*e −* 03, ****: *p <*= 1.00*e −* 04).

**Figure 6:**
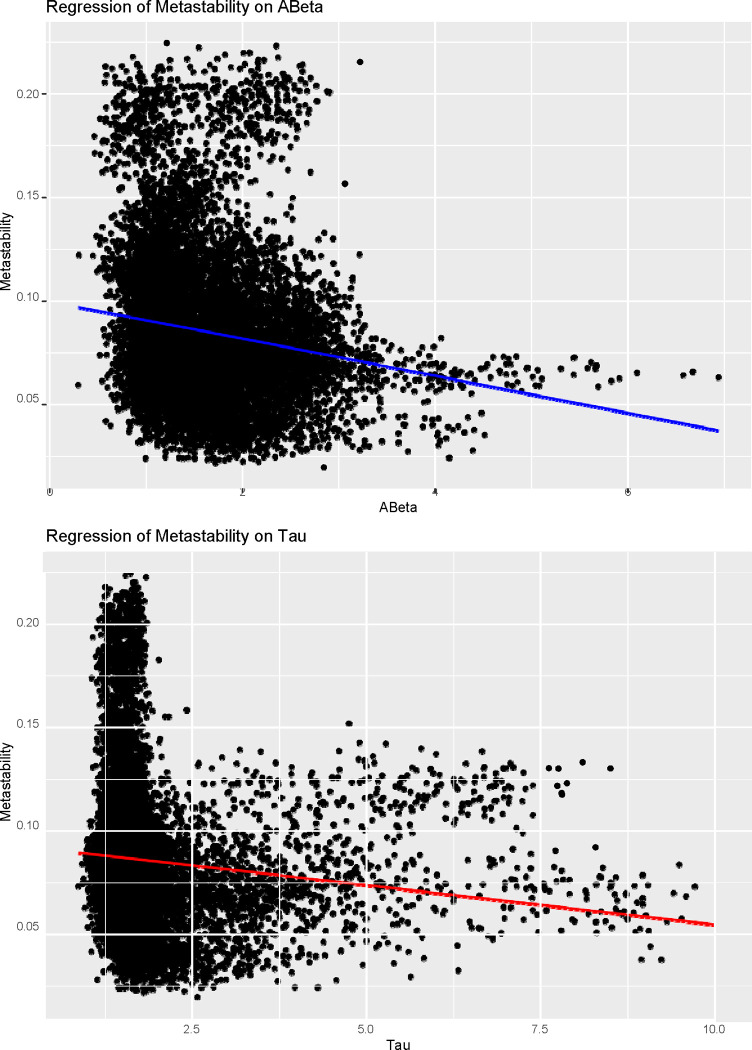
Multilevel models of the effects of ABeta and Tau on whole-brain metastability. Points represent the observed data, and lines represent the multilevel model-implied intercepts (mean of model coefficients for the random effect of subjects) and slopes (model coefficients for ABeta and tau). At the top panel, we can observe the effect of Abeta (in blue) on node-metastability in the whole brain. From the model, we know that the effect of Beta on node-metastability is significant (p = 0.014 *). At the bottom panels, in red, we have the effect of tau on node-metastability in the whole brain, which did not yield statistically significant results.

We assessed whether the outcome variable (i.e., each node’s metastability) shows a significant change by specifying the node ABeta and tau levels, as well as their interaction, as fixed effects, regardless of any other variable of the study. As we can observe from figures 5 and 6, that, as the disease progresses, there is a clear increase in the overall amount of ABeta and tau in the brain, while its metastability significantly decreases with both burdens. The analysis shows a significant dependence of the Metastability on ABeta (Estimate = 1.662e-03, Std. Error = 6.766e-04, p = 0.014 *). In this general analysis, tau and the interaction between both proteins did not play a significant role. The information we obtain from these results can be refined by doing a network-based metastability analysis, as done next.

#### Resting State Networks:

**Figure 7:**
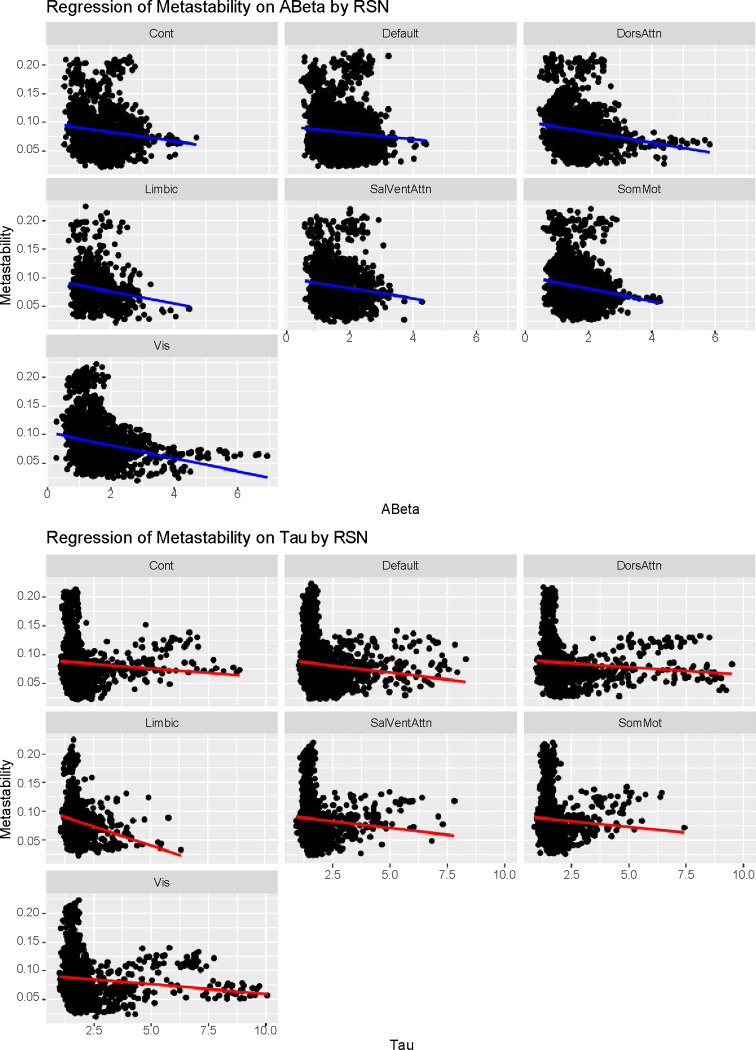
Multilevel models of the effects of ABeta and Tau on the Resting State Networks (RSN). At the top panels, we plot the multilevel model-implied intercepts and slopes of the ABeta on metastability (blue), which did not yield significant results. However, the effect of tau (red) is significant for both the Dorsal Attention network (p = 0.00819 **) and the somatomotor network (p = 0.04699 *). but not on the other networks. Finally, although not plotted, our analysis showed that the ABeta-tau interaction terms have a direct impact mainly on Dorsal Attention network (p = 0.01348 *), showing the toxic feedback loop between both burdens on the disease evolution [8].

Then we repeated the previous analysis considering the RSN for each node. Here, we can appreciate that the role of tau is significant for both the Dorsal Attention network (Estimate = 6.242e-03, Std. Error = 2.339e-03, p = 0.00819 **) and the somatomotor network (Estimate = 7.007e-03, Std. Error = 3.519e-03, p = 0.04699 *). We also see that the ABeta-tau interaction terms have a direct impact mainly on Dorsal Attention network (Estimate = −1.978e-03, Std. Error = 7.970e-04, p = 0.01348 *), showing the synergistic effect of both burdens on the disease evolution [8]. See Figure 7

## 4 Discussion

This study investigated the dynamical complexity underlying Alzheimer’s disease progression (i.e., over healthy controls, MCI, and AD). First, healthy controls presented the highest metastability values across the whole-brain functional network, followed by MCI and AD stages. Furthermore, our results revealed that resting-state networks vary significantly according to the disease stage. Finally, we used mixed effects to assess the impact of Amyloid-beta and tau, the two hallmark misfolded proteins related to the disease progression, on brain dynamics. We found that both significantly impact the whole-brain functional network and, in general, all resting-state networks. The damage progressively impairs brain function, leaving relatively untouched the visual, dorsal attentional, and somatomotor networks in earlier stages (i.e., MCI) but showing a general decline in later stages (i.e., AD).

At the whole-brain network, we found that the dynamical complexity showed the highest variability across time (i.e., node-metastability) for healthy controls, followed by progressive degradation for MCI. Furthermore, as individuals progressed from MCI to AD, we observed a clear decrease in node-metastability, indicating a gradual deterioration in brain network dynamics (Figure 2A). This sequential trajectory provides valuable insights into the impact of neurodegeneration on whole-brain dynamic changes and the progression from health to disease. Only a few works analyzed the impact of AD on whole-brain dynamics and information processing across large-scale brain networks. Sanz-Arigita et al. [39] did a graph analysis of the fMRI resting state functional connectivity and found that the empirical data pointed to increased synchronization of frontal cortices, together with a clear decrease at the parietal and occipital areas, which results in a net reduction of functional long-distance links between frontal and caudal brain regions. Wu et al. [48] found similar results using group information-guided ICA, showing that rs-FC alterations mostly appear in the temporal, cingulate, and angular areas. Mohammadian et al. [30] used the effective connectivity computed with spectral dynamic causal modeling analysis to compute the information flow within RSN, finding that impaired flow and disrupted causal interaction were found in amnesic MCI and AD groups with respect to healthy controls.

Recent resting-state fMRI enables exploring the brain’s intrinsic organization of large-scale distributed networks, revealing that Alzheimer’s disease modulates brain dynamics in resting-state networks, including a strong reduction in functional connectivity in the DMN, salience and subcortical networks during the initial stages [3, 6, 18, 22, 25, 33, 35, 38, 41]. Our results revealed node-metastability disease-dependent changes across large-scale resting-state networks (Figure 2). In particular, we found that the visual network exhibited the highest metastability values during all stages, remaining relatively unchanged in MCI to controls but showing a severe decrease in later AD stages. In general, we observe a significant reduction in the metastability of all networks along the disease progression, the visual network being the one that lasts longer without being affected, although it is altered anyway. Other networks, such as dorsal attentional and somatomotor networks, also follow this disruptive pattern but display a decrease in metastability from the disease’s earliest stages. Overall, these results demonstrate Alzheimer’s Disease-related changes in large-scale brain networks.

Sorg and coauthors [41], in one of the first works analyzing the resting-state networks, found that some areas within the DMN and the attention networks showed reduced activity in amnesic MCI to healthy controls. At the same time, other regions remain relatively unaffected, which is a result that matches our findings here. Brier et al. [6] set out to study the effect of AD on iter- and intra-resting state network connectivity in subjects ranging from healthy, very mild MCI and MCI (categorized according to their clinical dementia rating, CDR), observing a loss of correlation within the DMN and other networks even at very mild MCI. Also, they observed reduced correlations for all networks in MCI, while cross-network correlations were also reduced with disease severity, a result verified by our findings here. Li et al. [25] used Bayesian Network learning to determine the loss of connectivity between the different networks of AD patients to healthy controls. Also, they observed a serious reduction in the Integration degree measured for the DMN. Following this line, Badhwar and coauthors [3] did a review and meta-analysis of the AD literature, observing consistent alterations mainly in the DMN, salience, and limbic networks. These results have been confirmed by our study, where node-metastability also showed a consistent decrease in network activity, while our *HDF* index also reflects the decrease in the hierarchical structure of the brain in AD.

Later, Puttaert et al [33] used magnetoencephalography (MEG) and PET to study the alterations on the RSN caused along the AD continuum, finding that the only significant group effect was on the DMN for the mean lifetime and the functional occupancy of the 8 transient recurrent states in which they grouped the fMRI time-series. Given these results, Ibrahim et al. [22] performed a systematic literature review to determine the power of fMRI as a diagnostic tool in detecting network connectivity. Their main conclusion was that FC based on rs-fMRI was an excellent tool for diagnosing AD, mainly in the DMN. Rauchmann and collaborators [35] further confirmed these results by correlating global network integration measures with the clinical and cognitive subject performance. Rosas et al. [38] Ghahremani et al. [18] Our study extends these results by showing the high discriminating power of the ignition framework, in particular the metastability values, to discern the different disease stages, as shown in Figure 2.

Buckner et al. [7] studied the relationship between the DMN, memory, and ABeta activity pattern, finding a high correlation between the DMN activity pattern and the topography of ABeta deposition in elderly AD patients. Ingala et al. [23] further analyzed this relationship in cognitively healthy individuals, finding an inverse relation between ABeta levels and FC in the DMN, both for PET-measured and cerebrospinal fluid (CSF) ABeta levels in the posterior cingulate cortex (PCC), but not in other DMN areas, thus explaining not only the systematic negative slope estimate we found for ABeta, but also the lack of statistical significance in our results, as our measure for the DMN represents an aggregate of all the areas in that network. Such studies have shown that AD and burden (i.e., ABeta and tau) concentrations impact whole-brain dynamics. However, they presented some limitations. For example, brain activity patterns can vary across disease stages influenced by different factors such as age, comorbidity diseases with their respective treatments, or different treatments for removal of ABeta (e.g., with Adacanumab and Lecanemab), which are currently discussed in light of inconclusive effects on reducing cognitive decline [1]. Our investigation aligns and extends these findings by examining a cohort of 36 subjects during three stages: controls, MCI, and AD. In particular, we assessed mixed-effects models to examine the effects of Abeta and tau on node-metastability across the whole-brain and resting state networks. Our results revealed that both proteins significantly decrease whole-brain metastability. Also, the high sensitivity of metastability allowed us to conjecture the empirical observation of the synergistic effect of ABeta and tau in the last stages of AD. Furthermore, we observed burden-related effects in node-metastability in all the networks, with some exceptions (e.g., limbic network). Our results are consistent with previous studies reporting a consistent decrease in network efficiency throughout the disease. Moreover, we found that ABeta has a significant role in MCI, especially in the salience and control networks. On the other hand, tau shows a dominant role in the later stages of the disease on the salience, control, and default mode networks. Also, at the AD stage, it significantly impacts the dorsal attention and somatomotor networks. Finally, we observe that their interaction directly impacts all RSNs except the limbic one, starting in MSI up to the AD stage, showing the synergistic effect of both burdens on the disease evolution [8].

In summary, our research has important implications for our understanding of the effects of Alzheimer’s disease on brain dynamics. This study demonstrates that the stage of the disease strongly affects the dynamic complexity of the whole-brain functional network and large-scale resting-state networks. This research may have implications for elucidating the effects of Amyloid-beta and tau on cognition, mood, and behavior in mental disorders and dementia-related disorders.

## Figures and Tables

**Table 1:** Epidemiological information of the population used in this study.

Diagnosis	n (female)	Mean age	*σ*	Min. age	Max. age	Mean MMSE	*σ_MMSE_*	Min. MMSE	Max. MMSE	Mean ABeta	Mean tau

HC	17 (10)	70.8	4.3	63.1	78.0	29.3	0.7	28	30	1.31	1.53
MCI	9 (3)	68.8	5.8	57.8	76.6	27.4	1.5	25	30	1.52	1.80
AD	10 (5)	72.0	9.6	55.9	86.1	21.3	6.8	9	30	2.01	2.46

## Data Availability

Upon acceptance, all code for implementing computational models and reproducing our results will be available at https://github.com/dagush/WholeBrain

## Research-in-Context

### Systematic review:

The authors searched the scientific literature using traditional bibliographic indexing systems, such as PubMed and Google Scholar. Although there have been approaches that assess the impact of a single burden in AD, namely Amyloid-Beta (ABeta) on neuronal dynamics, which have been appropriately cited, there have been no approaches studying the combined effect of both ABeta and tau in light of the Intrinsic Ignition Framework, which allows to discern their weights as well as their combined effect on the brain and the resting state networks.

### Interpretation:

The main objective of this paper is to use whole-brain modeling techniques, particularly the Intrinsic Ignition Framework, to study the impact of both Abeta and tau on the dynamics of regional behaviors. As such, we used our results to discern the effect of each protein in isolation and combination, being able to assess their relative weights on contributing to abnormal brain activity and their affectation on the different Resting State Networks throughout the disease evolution.

### Future directions:

The study shows that whole-brain modeling enables research on disease mechanisms in vivo, demonstrating its potential to produce improved diagnostics and help discover new therapies (e.g., by simulating novel treatments).

## Footnotes

1
https://surfer.nmr.mgh.harvard.edu/fswiki/FreeSurferMethodsCitation

2
https://www.humanconnectome.org/software/connectome-workbench
