# Supplementary material for: Altered intrinsic ignition dynamics linked to Amyloid-β and tau pathology in Alzheimer’s disease
Source: bioRxiv. 2025 May 11:2024.03.29.587333. Originally published 2024 Mar 31. Preprint. [Version 2] doi: 10.1101/2024.03.29.587333 (PMC10996678; doi:10.1101/2024.03.29.587333)
Supplement: Supplement 1 [file media-1.pdf]

Understanding the Influence of Amyloid-Beta and Tau in Alzheimer's Disease through Whole-Brain Dynamics

Gustavo Patow[1,2,#], Anira Escrichs[2], Petra Ritter[3], Gustavo Deco[2,4], for the Alzheimer's Disease Neuroimaging Initiative

SUPPLEMENTARY MATERIAL

Supplementary Table 1. MPRAGE metadata.

ID	Model	TE [ms]	TR [s]	MatrixSize	VoxelSize [mm]
023_S_1190	TrioTim	2.98	2.3	(176, 240, 256)	(1.0, 1.0, 1.0)
002_S_1280	Prisma_fit	2.95	2.3	(176, 240, 256)	(1.2000046, 1.0546875, 1.0546875)
011_S_4547	Prisma_fit	2.98	2.3	(208, 240, 256)	(1.0, 1.0, 1.0)
168_S_6142	Prisma_fit	2.98	2.3	(208, 240, 256)	(0.9999948, 1.0, 1.0)
002_S_6103	Prisma_fit	2.98	2.3	(208, 240, 256)	(1.0, 1.0, 1.0)
002_S_4654	Prisma_fit	2.95	2.3	(176, 240, 256)	(1.199997, 1.0546875, 1.0546875)
022_S_5004	TrioTim	2.98	2.3	(176, 240, 256)	(1.0, 1.0, 1.0)
003_S_6067	Prisma	2.98	2.3	(208, 240, 256)	(1.0, 1.0, 1.0)
002_S_4229	Prisma_fit	2.98	2.3	(208, 240, 256)	(1.0, 1.0, 1.0)
012_S_6073	Prisma	2.98	2.3	(208, 240, 256)	(1.0, 1.0, 1.0)
002_S_1261	Prisma_fit	2.95	2.3	(176, 240, 256)	(1.2000046, 1.0546875, 1.0546875)
002_S_6009	Prisma_fit	2.95	2.3	(176, 240, 256)	(1.2000046, 1.0546875, 1.0546875)
007_S_4488	Prisma	2.98	2.3	(208, 240, 256)	(1.0, 1.0, 1.0)
003_S_4288	Prisma	2.98	2.3	(208, 240, 256)	(1.0, 1.0, 1.0)
002_S_4213	Prisma_fit	2.98	2.3	(208, 240, 256)	(1.0, 1.0, 1.0)
114_S_6039	Verio	2.98	2.3	(176, 240, 256)	(1.0, 1.0, 1.0)
036_S_4430	Skyra	2.95	2.3	(176, 240, 256)	(1.199997, 1.0546875, 1.0546875)
041_S_4974	Prisma_fit	2.95	2.3	(176, 240, 256)	(1.2000046, 1.0546875, 1.0546875)
007_S_4272	Prisma	2.98	2.3	(208, 240, 256)	(1.0, 1.0, 1.0)
011_S_4827	Prisma_fit	2.98	2.3	(208, 240, 256)	(1.0, 1.0, 1.0)
002_S_6053	Prisma_fit	2.98	2.3	(208, 240, 256)	(1.0, 1.0, 1.0)
003_S_4644	Prisma	2.98	2.3	(208, 240, 256)	(1.0, 1.0, 1.0)
002_S_4799	Prisma_fit	2.95	2.3	(176, 240, 256)	(1.2000046, 1.0546875, 1.0546875)
002_S_0413	Prisma_fit	2.95	2.3	(176, 240, 256)	(1.2000046, 1.0546875, 1.0546875)
114_S_0416	Verio	2.98	2.3	(176, 240, 256)	(1.0, 1.0, 1.0)
002_S_5178	Prisma_fit	2.95	2.3	(176, 240, 256)	(1.199997, 1.0546875, 1.0546875)
002_S_6030	Prisma_fit	2.95	2.3	(176, 240, 256)	(1.2000046, 1.0546875, 1.0546875)
003_S_1122	Prisma	2.98	2.3	(208, 240, 256)	(1.0000056, 1.0, 1.0)
011_S_4893	Prisma_fit	2.98	2.3	(208, 240, 256)	(1.0, 1.0, 1.0)

002_S_1155	Prisma_fit	2.95	2.3	(176, 240, 256)	(1.2000046, 1.0546875, 1.0546875)
036_S_4715	Skyra	2.95	2.3	(176, 240, 256)	(1.2000046, 1.0546875, 1.0546875)
007_S_4387	Prisma	2.98	2.3	(208, 240, 256)	(1.0, 1.0, 1.0)
007_S_4620	Prisma	2.98	2.3	(208, 240, 256)	(1.0, 1.0, 1.0)

Supplementary Table 2. FLAIR metadata.

ID	Model	TE [ms]	TR [s]	MatrixSize	VoxelSize [mm]
023_S_1190	TrioTim	443	4.8	(160, 256, 256)	(1.2000046, 1.0, 1.0)
002_S_1280	Prisma_fit	441	4.8	(160, 256, 256)	(1.2000046, 1.0, 1.0)
011_S_4547	Prisma_fit	441	4.8	(160, 256, 256)	(1.2000046, 1.0, 1.0)
168_S_6142	Prisma_fit	441	4.8	(160, 256, 256)	(1.2000002, 1.0, 1.0)
002_S_6103	Prisma_fit	441	4.8	(160, 256, 256)	(1.199997, 1.0, 1.0)
002_S_4654	Prisma_fit	441	4.8	(160, 256, 256)	(1.199997, 1.0, 1.0)
022_S_5004	TrioTim	439	4.8	(160, 256, 256)	(1.199997, 1.0, 1.0)
003_S_6067	Prisma	441	4.8	(160, 256, 256)	(1.2000046, 1.0, 1.0)
002_S_4229	Prisma_fit	441	4.8	(160, 256, 256)	(1.199997, 1.0, 1.0)
012_S_6073	Prisma	441	4.8	(160, 256, 256)	(1.2000046, 1.0, 1.0)
002_S_1261	Prisma_fit	441	4.8	(160, 256, 256)	(1.2000046, 1.0, 1.0)
002_S_6009	Prisma_fit	441	4.8	(160, 256, 256)	(1.2000046, 1.0, 1.0)
007_S_4488	Prisma	441	4.8	(160, 256, 256)	(1.2000046, 1.0, 1.0)
003_S_4288	Prisma	441	4.8	(160, 256, 256)	(1.199997, 1.0, 1.0)
002_S_4213	Prisma_fit	441	4.8	(160, 256, 256)	(1.199997, 1.0, 1.0)
114_S_6039	Verio	343	4.8	(160, 256, 256)	(1.0, 1.0, 1.0)
036_S_4430	Skyra	441	4.8	(160, 256, 256)	(1.2000046, 1.0, 1.0)
041_S_4974	Prisma_fit	441	4.8	(160, 256, 256)	(1.199997, 1.0, 1.0)
007_S_4272	Prisma	441	4.8	(160, 256, 256)	(1.199997, 1.0, 1.0)
011_S_4827	Prisma_fit	441	4.8	(160, 256, 256)	(1.199997, 1.0, 1.0)
002_S_6053	Prisma_fit	441	4.8	(160, 256, 256)	(1.199997, 1.0, 1.0)
003_S_4644	Prisma	441	4.8	(160, 256, 256)	(1.199997, 1.0, 1.0)
002_S_4799	Prisma_fit	441	4.8	(160, 256, 256)	(1.2000046, 1.0, 1.0)
002_S_0413	Prisma_fit	441	4.8	(160, 256, 256)	(1.2000046, 1.0, 1.0)
114_S_0416	Verio	343	4.8	(160, 256, 256)	(1.0, 1.0, 1.0)
002_S_5178	Prisma_fit	441	4.8	(160, 256, 256)	(1.199997, 1.0, 1.0)
002_S_6030	Prisma_fit	441	4.8	(160, 256, 256)	(1.2000046, 1.0, 1.0)
003_S_1122	Prisma	441	4.8	(160, 256, 256)	(1.2000005, 1.0, 1.0)
011_S_4893	Prisma_fit	441	4.8	(160, 256, 256)	(1.2000046, 1.0, 1.0)
002_S_1155	Prisma_fit	441	4.8	(160, 256, 256)	(1.2000046, 1.0, 1.0)
036_S_4715	Skyra	441	4.8	(160, 256, 256)	(1.2000046, 1.0, 1.0)
007_S_4387	Prisma	441	4.8	(160, 256, 256)	(1.2000046, 1.0, 1.0)
007_S_4620	Prisma	441	4.8	(160, 256, 256)	(1.2000046, 1.0, 1.0)

Supplementary Table 3. DTI metadata (only for HC participants to average the SC template)

ID	Model	Institute	TE [ms]	TR [s]	MatrixSize	VoxelSize [mm, mm, mm, s]	n_Bvec s	Bvals
002_S_1280	Prisma_fit	OHSU_AIRC	56	7.2	(116, 116, 80,55)	(2.0, 2.0, 2.0, 7.2)	49	[0. 1000.]
002_S_6103	Prisma_fit	OHSU_AIRC	56	7.2	(116, 116, 80,55)	(2.0, 2.0, 2.0, 7.2)	49	[0. 1000.]
003_S_6067	Prisma	USCINI	56	7.2	(116, 116, 80,55)	(2.0, 2.0, 2.0, 7.2)	49	[0. 1000.]
002_S_6009	Prisma_fit	OHSU_AIRC	56	7.2	(116, 116, 80,55)	(2.0, 2.0, 2.0, 7.2)	49	[0. 1000.]
007_S_4488	Prisma	MAYO_CLINIC_MRI_58	71	3.4	(116, 116, 81,127)	(2.0, 2.0, 2.0, 3.4)	115	[0. 500. 1000. 2000.]
003_S_4288	Prisma	USC_Stevens_Hall_Institut e	56	7.2	(116, 116, 80,55)	(2.0, 2.0, 2.0, 7.2)	49	[0. 1000.]
002_S_4213	Prisma_fit	OHSU_AIRC	56	7.2	(116, 116, 80,55)	(2.5172415, 2.5172415, 2.0, 7.2)	49	[0. 1000.]
002_S_6053	Prisma_fit	OHSU_AIRC	56	7.2	(116, 116, 80,55)	(2.0, 2.0, 2.0, 7.2)	49	[0. 1000.]
003_S_4644	Prisma	USCINI	56	7.2	(116, 116, 80,55)	(2.0, 2.0, 2.0, 7.2)	49	[0. 1000.]
002_S_4799	Prisma_fit	OHSU_AIRC	56	7.2	(116, 116, 80,55)	(2.0, 2.0, 2.0, 7.2)	49	[0. 1000.]
002_S_0413	Prisma_fit	OHSU_AIRC	56	7.2	(116, 116, 80,55)	(2.0, 2.0, 2.0, 7.2)	49	[0. 1000.]
002_S_5178	Prisma_fit	OHSU_AIRC	56	7.2	(116, 116, 80,55)	(2.0, 2.0, 2.0, 7.2)	49	[0. 1000.]
002_S_6030	Prisma_fit	OHSU_AIRC	56	7.2	(116, 116, 80,55)	(2.0, 2.0, 2.0, 7.2)	49	[0. 1000.]
007_S_4387	Prisma	MAYO_CLINIC_MRI_58	71	3.4	(116, 116, 81,127)	(2.0, 2.0, 2.0, 3.4)	115	[0. 500. 1000. 2000.]
007_S_4620	Prisma	MAYO_CLINIC_MRI_58	71	3.4	(116, 116, 81,127)	(2.0, 2.0, 2.0, 3.4)	115	[0. 500. 1000. 2000.]

Supplementary Table 4. AV-45 PET (Abeta) metadata.

ID	Scanner	Model	MatrixSize	VoxelSize [mm]
023_S_1190	Siemens	Biograph6_TruePoint	(160, 160, 96)	(1.5, 1.5, 1.5)
002_S_1280	Philips	GEMINI_TF_TOF_16	(160, 160, 96)	(1.5, 1.5, 1.5)
011_S_4547	Siemens	Biograph40_TruePoint	(160, 160, 96)	(1.5, 1.5, 1.5)
168_S_6142	GE	Discovery_STE	(160, 160, 96)	(1.5, 1.5, 1.5)
002_S_6103	Philips	GEMINI_TF_TOF_16	(160, 160, 96)	(1.5, 1.5, 1.5)
002_S_4654	Philips	GEMINI_TF_TOF_16	(160, 160, 96)	(1.5, 1.5, 1.5)
022_S_5004	Philips	Ingenuity_TF_PET_CT	(160, 160, 96)	(1.5, 1.5, 1.5)
003_S_6067	Siemens	Biograph64_TruePoint	(160, 160, 96)	(1.5, 1.5, 1.5)
002_S_4229	Philips	GEMINI_TF_TOF_16	(160, 160, 96)	(1.5, 1.5, 1.5)
012_S_6073	GE	Discovery_710	(160, 160, 96)	(1.5, 1.5, 1.5)
002_S_1261	Philips	GEMINI_TF_TOF_16	(160, 160, 96)	(1.5, 1.5, 1.5)
002_S_6009	Philips	GEMINI_TF_TOF_16	(160, 160, 96)	(1.5, 1.5, 1.5)
007_S_4488	GE	Discovery_690	(160, 160, 96)	(1.5, 1.5, 1.5)
003_S_4288	Siemens	Biograph64_TruePoint	(160, 160, 96)	(1.5, 1.5, 1.5)
002_S_4213	Philips	GEMINI_TF_TOF_16	(160, 160, 96)	(1.5, 1.5, 1.5)
114_S_6039	Philips	GEMINI_TF_TOF_64	(160, 160, 96)	(1.5, 1.5, 1.5)
036_S_4430	Siemens	Biograph40_TruePoint	(160, 160, 96)	(1.5, 1.5, 1.5)
041_S_4974	Siemens	HR+	(160, 160, 96)	(1.5, 1.5, 1.5)
007_S_4272	GE	Discovery_690	(160, 160, 96)	(1.5, 1.5, 1.5)
011_S_4827	Siemens	Biograph40_TruePoint	(160, 160, 96)	(1.5, 1.5, 1.5)
002_S_6053	Philips	GEMINI_TF_TOF_16	(160, 160, 96)	(1.5, 1.5, 1.5)
003_S_4644	Siemens	Biograph64_TruePoint	(160, 160, 96)	(1.5, 1.5, 1.5)
002_S_4799	Philips	GEMINI_TF_TOF_16	(160, 160, 96)	(1.5, 1.5, 1.5)
002_S_0413	Philips	GEMINI_TF_TOF_16	(160, 160, 96)	(1.5, 1.5, 1.5)
114_S_0416	Philips	GEMINI_TF_TOF_64	(160, 160, 96)	(1.5, 1.5, 1.5)
002_S_5178	Philips	GEMINI_TF_TOF_16	(160, 160, 96)	(1.5, 1.5, 1.5)
002_S_6030	Philips	GEMINI_TF_TOF_16	(160, 160, 96)	(1.5, 1.5, 1.5)
003_S_1122	Siemens	Biograph64_TruePoint	(160, 160, 96)	(1.5, 1.5, 1.5)
011_S_4893	Siemens	Biograph40_TruePoint	(160, 160, 96)	(1.5, 1.5, 1.5)
002_S_1155	Philips	GEMINI_TF_TOF_16	(160, 160, 96)	(1.5, 1.5, 1.5)
036_S_4715	Siemens	Biograph40_TruePoint	(160, 160, 96)	(1.5, 1.5, 1.5)
007_S_4387	GE	Discovery_690	(160, 160, 96)	(1.5, 1.5, 1.5)
007_S_4620	GE	Discovery_690	(160, 160, 96)	(1.5, 1.5, 1.5)

Supplementary Table 5. AV-14-51 PET (Tau) metadata

ID	Scanner	Model	MatrixSize	VoxelSize [mm]
023_S_1190	Siemens	Biograph6_TruePoint	(160, 160, 96)	(1.5, 1.5, 1.5)
002_S_1280	Philips	GEMINI_TF_TOF_16	(160, 160, 96)	(1.5, 1.5, 1.5)
011_S_4547	Siemens	Biograph40_TruePoint	(160, 160, 96)	(1.5, 1.5, 1.5)
168_S_6142	GE	Discovery_STE	(160, 160, 96)	(1.5, 1.5, 1.5)
002_S_6103	Philips	GEMINI_TF_TOF_16	(160, 160, 96)	(1.5, 1.5, 1.5)
002_S_4654	Philips	GEMINI_TF_TOF_16	(160, 160, 96)	(1.5, 1.5, 1.5)
022_S_5004	Philips	Ingenuity_TF_PET_CT	(160, 160, 96)	(1.5, 1.5, 1.5)
003_S_6067	Siemens	Biograph64_TruePoint	(160, 160, 96)	(1.5, 1.5, 1.5)
002_S_4229	Philips	GEMINI_TF_TOF_16	(160, 160, 96)	(1.5, 1.5, 1.5)
012_S_6073	GE	Discovery_710	(160, 160, 96)	(1.5, 1.5, 1.5)
002_S_1261	Philips	GEMINI_TF_TOF_16	(160, 160, 96)	(1.5, 1.5, 1.5)
002_S_6009	Philips	GEMINI_TF_TOF_16	(160, 160, 96)	(1.5, 1.5, 1.5)
007_S_4488	GE	Discovery_690	(160, 160, 96)	(1.5, 1.5, 1.5)
003_S_4288	Siemens	Biograph64_TruePoint	(160, 160, 96)	(1.5, 1.5, 1.5)
002_S_4213	Philips	GEMINI_TF_TOF_16	(160, 160, 96)	(1.5, 1.5, 1.5)
114_S_6039	Philips	GEMINI_TF_TOF_64	(160, 160, 96)	(1.5, 1.5, 1.5)
036_S_4430	Siemens	Biograph40_TruePoint	(160, 160, 96)	(1.5, 1.5, 1.5)
041_S_4974	Siemens	HR+	(160, 160, 96)	(1.5, 1.5, 1.5)
007_S_4272	GE	Discovery_690	(160, 160, 96)	(1.5, 1.5, 1.5)
011_S_4827	Siemens	Biograph40_TruePoint	(160, 160, 96)	(1.5, 1.5, 1.5)
002_S_6053	Philips	GEMINI_TF_TOF_16	(160, 160, 96)	(1.5, 1.5, 1.5)
003_S_4644	Siemens	Biograph64_TruePoint	(160, 160, 96)	(1.5, 1.5, 1.5)
002_S_4799	Philips	GEMINI_TF_TOF_16	(160, 160, 96)	(1.5, 1.5, 1.5)
002_S_0413	Philips	GEMINI_TF_TOF_16	(160, 160, 96)	(1.5, 1.5, 1.5)
114_S_0416	Philips	GEMINI_TF_TOF_64	(160, 160, 96)	(1.5, 1.5, 1.5)
002_S_5178	Philips	GEMINI_TF_TOF_16	(160, 160, 96)	(1.5, 1.5, 1.5)
002_S_6030	Philips	GEMINI_TF_TOF_16	(160, 160, 96)	(1.5, 1.5, 1.5)
003_S_1122	Siemens	Biograph64_TruePoint	(160, 160, 96)	(1.5, 1.5, 1.5)
011_S_4893	Siemens	Biograph40_TruePoint	(160, 160, 96)	(1.5, 1.5, 1.5)
002_S_1155	Philips	GEMINI_TF_TOF_16	(160, 160, 96)	(1.5, 1.5, 1.5)
036_S_4715	Siemens	Biograph40_TruePoint	(160, 160, 96)	(1.5, 1.5, 1.5)
007_S_4387	GE	Discovery_690	(160, 160, 96)	(1.5, 1.5, 1.5)
007_S_4620	GE	Discovery_690	(160, 160, 96)	(1.5, 1.5, 1.5)

Supplementary Table 6. Dates of Imaging and MMSE.

ID	MMSE date	MPRAGE date	FLAIR date	DTI date	AV-45 PET date	AV-1451 PET date
023_S_1190	17/11/13	17/10/23	17/10/23		17/10/25	17/11/08
002_S_1280	18/3/7	17/3/13	17/3/13	17/3/13	17/3/02	18/3/5
011_S_4547	17/8/18	17/8/18	17/8/18		17/8/30	17/8/24
168_S_6142	17/12/5	17/12/18	17/12/18		18/1/17	18/1/3
002_S_6103	17/10/25	17/11/20	17/11/20	17/11/20	17/11/21	18/1/17
002_S_4654	18/5/15	17/5/3	17/5/3		17/5/2	18/5/22
022_S_5004	18/6/29	18/3/14	17/3/21		17/3/21	17/4/5
003_S_6067	17/12/4	17/8/18	17/8/18	17/8/18	17/10/13	17/10/18
002_S_4229	18/5/14	17/9/20	17/9/20		17/9/20	17/10/3
012_S_6073	17/9/18	17/9/22	17/9/22		17/10/12	17/10/11
002_S_1261	18/3/8	17/3/15	17/3/15		17/3/14	17/3/15
002_S_6009	17/4/1	17/4/17	17/4/17	17/4/17	17/5/16	17/5/15
007_S_4488	18/6/11	17/9/12	17/9/12	17/9/12	17/9/22	17/9/13
003_S_4288	17/10/2	17/10/3	17/10/3	17/10/3	17/10/3	18/2/22
002_S_4213	17/8/16	17/8/14	17/8/14	17/8/14	17/8/14	17/8/17
114_S_6039	17/8/10	17/7/21	17/7/21		17/8/24	17/10/4
036_S_4430	17/11/15	17/11/07	17/11/07		17/11/15	17/11/21
041_S_4974	17/10/30	17/10/5	17/10/5		17/8/24	17/10/12
007_S_4272	18/1/18	18/1/16	18/1/16		17/12/19	18/1/17
011_S_4827	17/8/24	17/8/31	17/8/31		17/8/28	17/9/7
002_S_6053	17/7/21	17/7/18	17/7/18	17/7/18	17/8/23	17/8/24
003_S_4644	17/6/26	17/6/21	17/6/21	17/6/21	18/2/28	18/4/17
002_S_4799	18/6/7	17/5/22	17/5/22	17/5/22	17/5/18	18/6/13
002_S_0413	17/6/16	17/6/21	17/6/21	17/6/21	17/6/15	17/6/21
114_S_0416	18/7/24	17/10/24	17/10/24		17/10/24	17/11/21
002_S_5178	17/6/6	17/5/31	17/5/31	17/5/31	17/6/5	17/5/31
002_S_6030	17/6/9	17/6/15	17/6/15	17/6/15	17/7/25	17/7/24
003_S_1122	18/7/25	17/5/18	17/5/18		17/8/8	17/8/10
011_S_4893	18/7/17	17/11/8	17/11/8		17/11/1	17/11/7
002_S_1155	18/5/9	17/4/24	17/4/24		17/4/20	17/4/24
036_S_4715	17/10/13	17/10/10	17/10/10		17/10/10	17/10/12
007_S_4387	17/10/31	17/11/1	17/11/1	17/11/1	17/10/24	17/11/29
007_S_4620	17/12/12	17/12/05	17/12/05	17/12/05	17/12/06	17/12/14
