# Supplementary material for: Altered intrinsic ignition dynamics linked to Amyloid-β and tau pathology in Alzheimer’s disease
Source: bioRxiv. 2025 May 11:2024.03.29.587333. Originally published 2024 Mar 31. Preprint. [Version 2] doi: 10.1101/2024.03.29.587333 (PMC10996678; doi:10.1101/2024.03.29.587333)
Supplement: Supplement 2 [file NIHPP2024.03.29.587333v2-supplement-2.pdf]

bioRxiv preprint doi: https://doi.org/10.1101/2024.03.29.587333; this version posted May 11, 2025. The copyright holder for this preprint (which was not certified by peer review) is the author/funder, who has granted bioRxiv a license to display the preprint in perpetuity. It is made available under aCC-BY 4.0 International license.

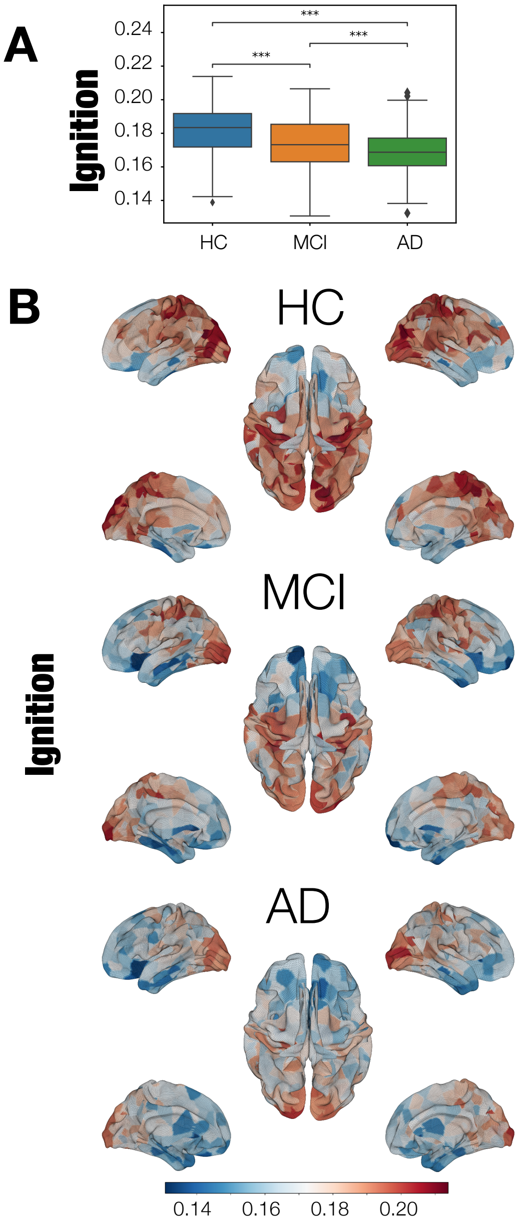


**FigS1.Intrinsic ignition is reduced across the AD continuum**. **(A)** Ignition. HCs showed higher ignition values at the whole-brain level compared to MCI and AD patients. P-values are based on a permutation test, where *** represents $p <= 1.00e-04$. **(B)** Brain renders represent the ignition values of the 379 areas for each AD stage.
